# Supplementary material for: Origin of circular and triangular pores in electron-irradiated hexagonal boron nitride
Source: Nat Commun. 2026 Aug 11;17:9620. doi: 10.1038/s41467-026-76612-2 (PMC13554199; doi:10.1038/s41467-026-76612-2)
Supplement: Supplementary file 1 [file 41467_2026_76612_MOESM1_ESM.pdf]

Supplementary Information

Origin of circular and triangular pores in electron-irradiated hexagonal boron nitride

Umair Javed[1,2], Manuel Längle[1], Vladimír Zobač[1], Alexander Markevich[1], Barbara Maria Mayer[1,3] Clara Kofler[1,2], Martin Paul[1], Darwin Lorber[1], Nandhini Ravindran[1,2], Clemens Mangler[1], Toma Susi[1], Jani Kotakoski[1]

[1] University of Vienna, Faculty of Physics, Boltzmanngasse 5, 1090 Vienna, Austria
[2] University of Vienna, Vienna Doctoral School in Physics, Boltzmanngasse 5, 1090 Vienna, Austria
[3] Uppsala University, Faculty of Physics, Box 516, 75120 Uppsala, Sweden

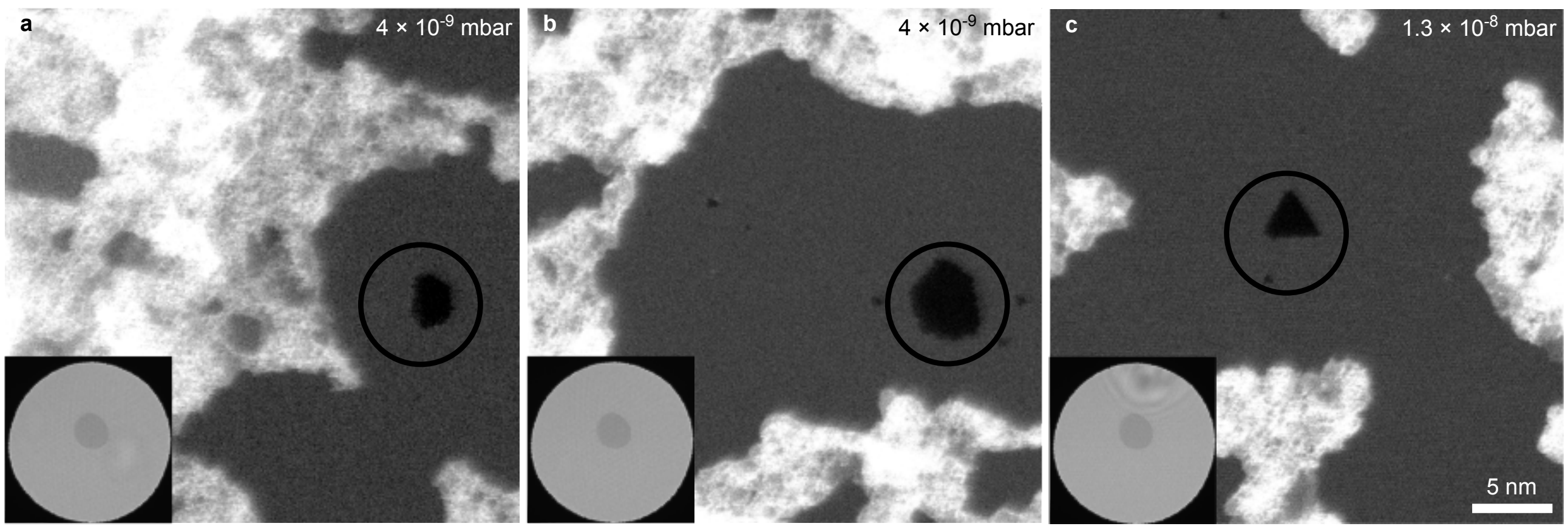

Supplementary Fig. 1. **Pore formation in parallel-beam (CCD) mode.** Different areas of hBN were irradiated for roughly 4 minutes with 60 kV electrons, a beam current of ca. 118 nA, with a defocus of 125 nm, and fluence of ca. 4.9×10^9 e/nm^2. (a-b) Oxygen partial pressure of 4×10^{-9} mbar. (c) Oxygen partial pressure of 1.3×10^{-8} mbar. The black circle roughly outlines the irradiated area, the insets in the lower-left corners show how the sample appeared on the CCD camera, and the scale bar corresponds to all the images. Even in parallel-beam mode, in an oxygen partial pressure in the range of low 10^{-8} mbar, the pores that emerge are not triangular, but when the pressure is increased to the 10^{-8} mbar range, the pores that are formed are triangular.

Supplementary Table 1. **The shapes of pores formed in parallel-beam mode (CCD), in different oxygen pressures, and at different defoci.** In these data, the pressure gauge reading at the objective area is used without any additional factor arising from the sample stage geometry (see the main text).

Pressure (mbar)	Defocus (nm)	$J\,(\mathrm{O_2 s^{-1} nm^{-2}})$	Dose ($\mathrm{e^- s^{-1} nm^{-2}}$)	Shape
4×10^{-9}	125	2	1.7×10^7	Non-triangle
4×10^{-9}	125	2	1.7×10^7	Non-triangle
4×10^{-9}	125	2	1.7×10^7	Non-triangle
4×10^{-9}	125	2	1.7×10^7	Non-triangle
4×10^{-9}	125	2	1.7×10^7	Non-triangle
1.3×10^{-8}	125	6	1.7×10^7	Triangle
4×10^{-9}	500	2	1×10^6	Triangle
4×10^{-9}	500	2	1×10^6	Triangle
4×10^{-9}	500	2	1×10^6	Triangle
1.3×10^{-8}	500	6	1×10^6	Triangle

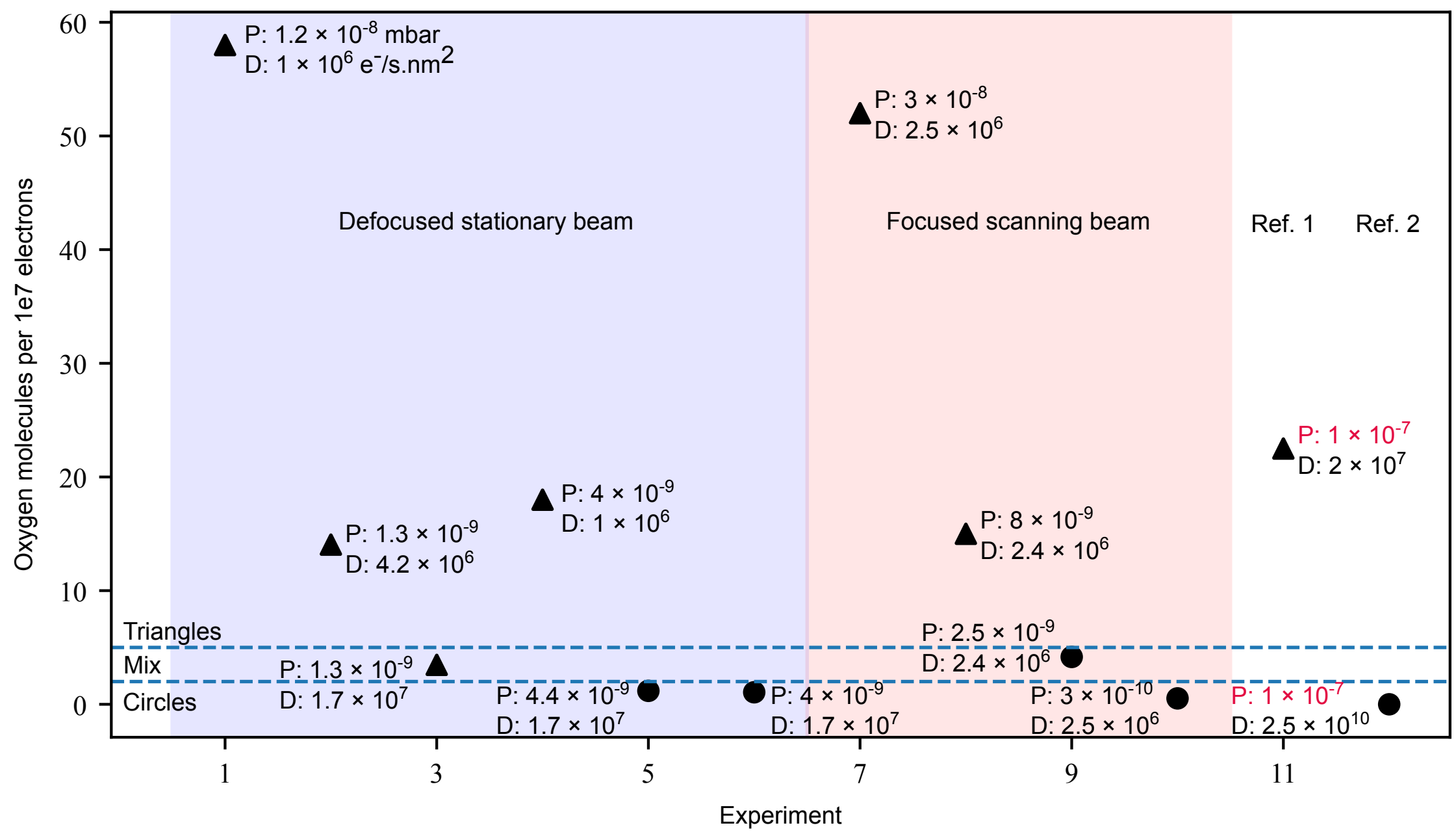

Supplementary Fig. 2. **Plot of the ratio of oxygen molecules impinging per 10 million electrons, and the pore shape in hBN at those conditions.** The horizontal axis shows different types of experiments, and the markers indicate whether a triangle or a circle corresponds to the pore shape. For comparison with studies in the literature, we used dose-rate values from Meyer et al. [1] and Keneipp et al. [2], and assumed a pressure of 1×10^{-7} mbar. In all data presented here, the pressure gauge reading at the objective area is used without any additional factor arising from the sample stage geometry (see the main text).

Supplementary Table 2. **Rate of Impinging.** Oxygen molecules impinging on the surface of the sample within a 16 nm^2 field of view, or an atomic site per frame, at each partial pressure (site here refers to a lattice site in a pristine hBN structure). In these data, a factor of 10 has been added to the impingement rate to account for the sample stage geometry (see the main text).

Pressure (10^{-8} mbar)	J ($10^{18}\mathrm{m}^{-2}\mathrm{s}^{-1}$)	J_{FOV}	J_{Site}
0.4	0.12	527	0.05
0.8	0.21	949	0.1
1.7	0.46	1992	0.2
2.5	0.68	2929	0.3
3.3	0.89	3867	0.4
4.1	1.1	4804	0.5
4.9	1.3	5742	0.6
5.7	1.5	6679	0.7
6.5	1.7	7617	0.8
7.3	1.9	8554	0.9
8.1	2.1	9492	1.0

Supplementary Table 3. **Total-energy difference between B–O–B and N–O–N configurations for different charge states.** Calculated for the double-vacancy defect in hBN with an oxygen atom (see the main article for the atomic configurations).

Charge (e^-)	ΔE (eV)
-2	-7.20
-1	-6.61
0	-3.70
+1	-3.10
+2	-3.07

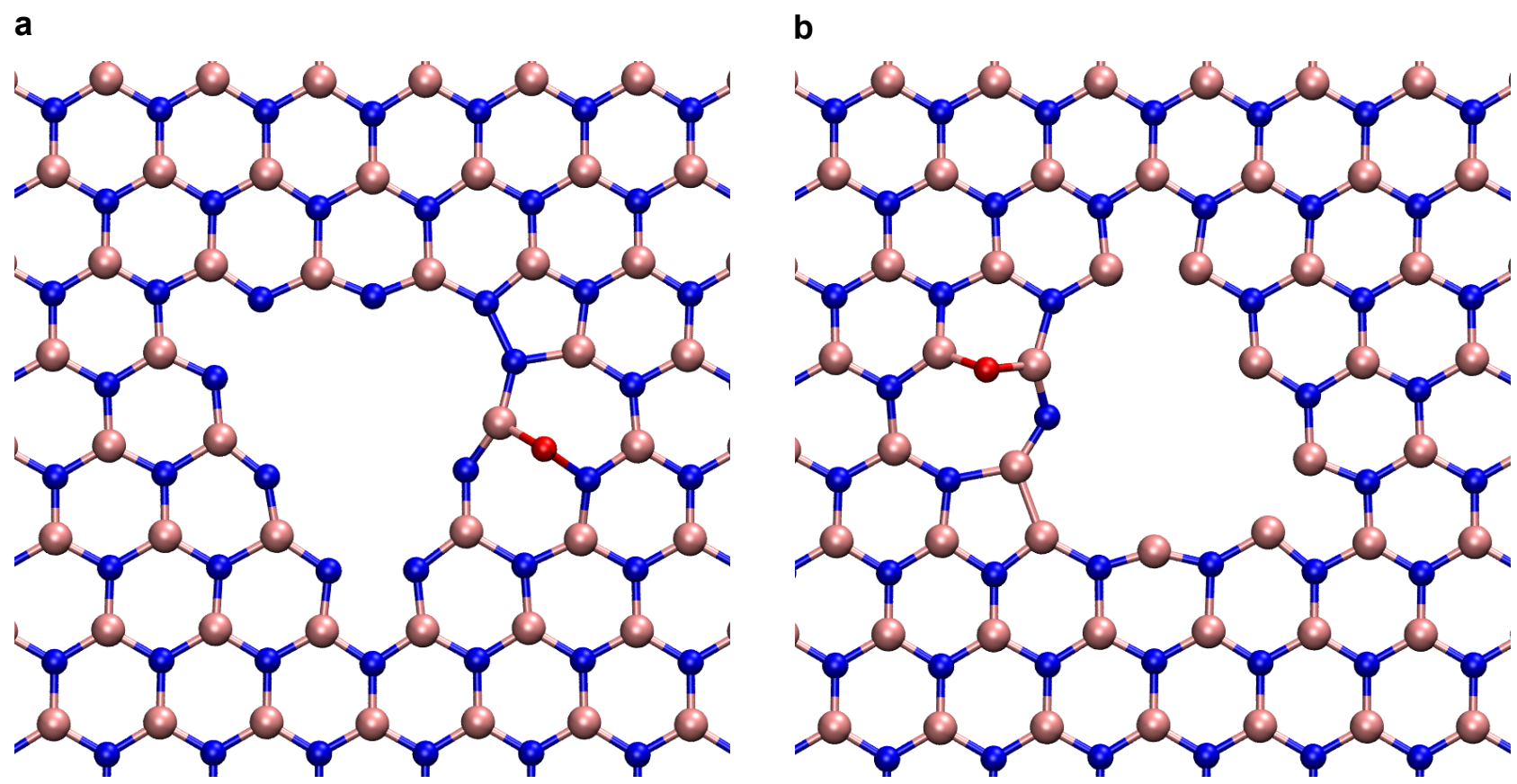

Supplementary Fig. 3. **Adsorption of oxygen on hBN near the edges of V_9 defects.** (a) In the case of V_9^N, oxygen radical adsorption on the B–N bond close to the edge leads to breaking of the bond (Fig. 6b), with the distance between B and N atoms becoming $\sim$2.5 Å. The adsorption energy is calculated to be -3.94 eV, which is significantly lower than that on the pristine surface. (b) Adsorption of O near the B-terminated edge leads to even more drastic changes in the bonding (Fig. 6c): the N atom is pushed towards the edge and a B–O–B bridge configuration is formed, resulting in the adsorption energy of -6.10 eV.

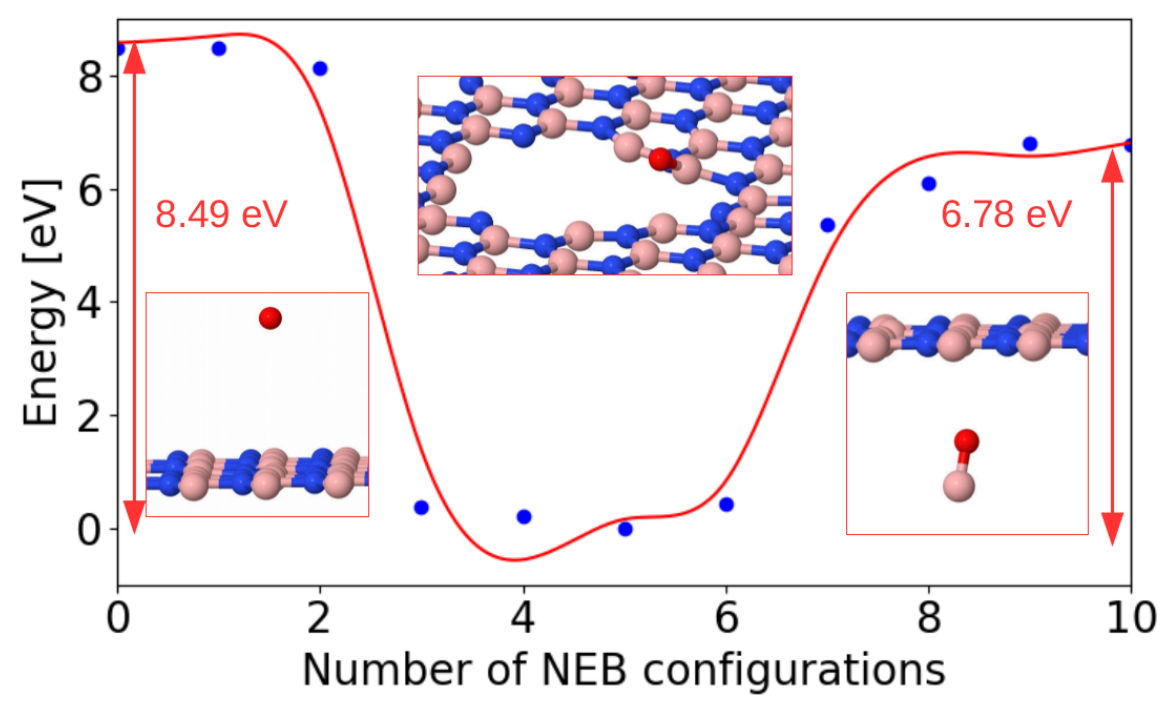

Supplementary Fig. 4. **Energy profile of the oxygen adsorption and desorption process on a boron-terminated $\mathbf{V}_9^{\mathrm{B}}$ edge obtained from NEB simulations.** The inset panels depict the initial, intermediate, and final configurations along the reaction path. The red curve serves as a visual guide, generated by spline interpolation of the discrete NEB energy points.

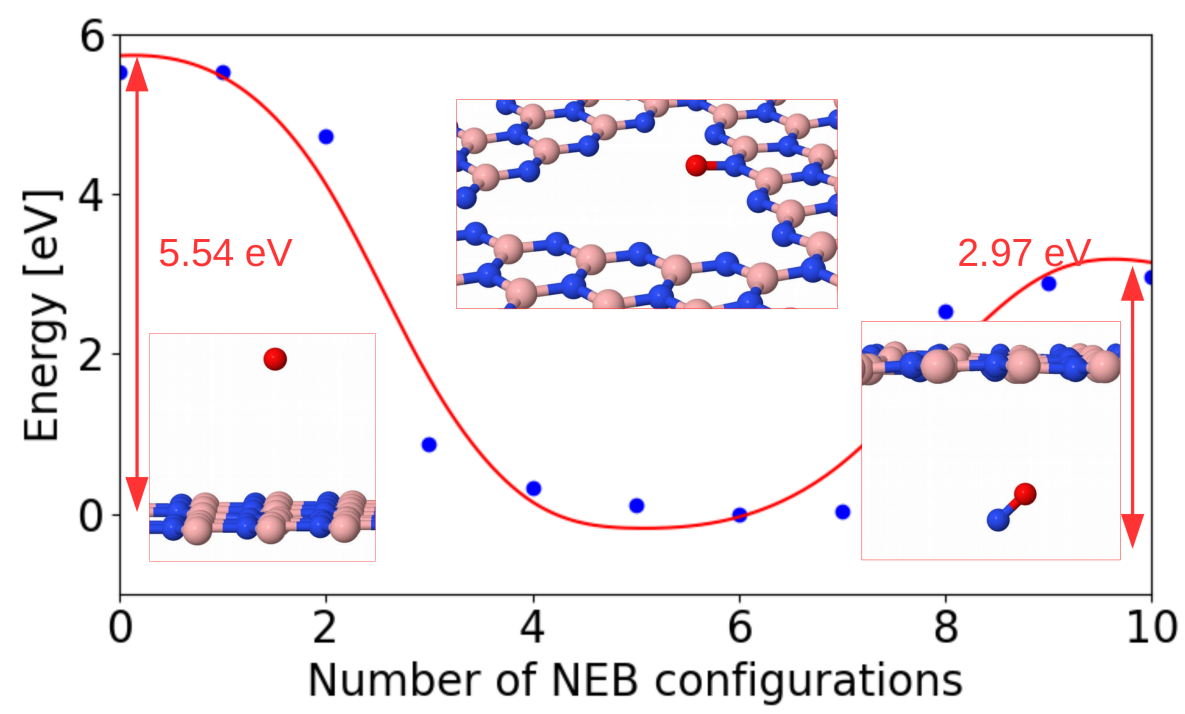

Supplementary Fig. 5. **Energy profile of the oxygen adsorption and desorption process on a nitrogen-terminated $\mathbf{V}_9^{\mathrm{N}}$ edge obtained from NEB simulations.** The inset panels depict the initial, intermediate, and final configurations along the reaction path. The red curve serves as a visual guide, generated by spline interpolation of the discrete NEB energy points.

[1] Jannik C. Meyer, Andrey Chuvilin, Gerardo Algara-Siller, Johannes Biskupek, and Ute Kaiser. Selective Sputtering and Atomic Resolution Imaging of Atomically Thin Boron Nitride Membranes. *Nano Letters*, 9(7):2683–2689, July 2009.
[2] Rachael N. Keneipp, Jordan A. Gusdorff, Pia Bhatia, Trey T. Shin, Lee C. Bassett, and Marija Drndić. Nanoscale Sculpting of Hexagonal Boron Nitride with an Electron Beam. *The Journal of Physical Chemistry C*, 128(21):8741–8749, May 2024.
