# Supplementary material for: Origin of circular and triangular pores in electron-irradiated hexagonal boron nitride
Source: Nat Commun. 2026 Aug 11;17:9620. doi: 10.1038/s41467-026-76612-2 (PMC13554199; doi:10.1038/s41467-026-76612-2)
Supplement: Supplementary file 2 [file 41467_2026_76612_MOESM2_ESM.pdf]

nature portfolio

Peer Review File

Origin of circular and triangular pores in electron-irradiated hexagonal boron nitride

Corresponding Author: Professor Jani Kotakoski

This file contains all reviewer reports in order by version, followed by all author rebuttals in order by version.

Version 0:

Reviewer comments:

Reviewer #1

(Remarks to the Author)
The manuscript presents experimental evidence that oxygen radicals in the microscope column are responsible for the formation of triangular pores in hBN under electron irradiation. The systematic control of oxygen partial pressure and the quantitative analysis of edge atom removal probabilities represent clear strengths of the work. The results challenge a widely accepted interpretation in the previous articles. Nevertheless, several aspects of the mechanistic interpretation require clarification or further support before the conclusions can be considered robust. I list below several major points that should be addressed in a revised version.

Major comments
1. It is well known that under high-vacuum conditions in TEM, triangular hole formation with N-termination can be clearly observed because 80 kV lies between the knock-on threshold values of B (74 kV) and N (84 kV)1.[Ref.1] While the present manuscript tried to demonstrate that oxygen plays a key role in determining the pore shape, I think the difference between the presence and absence of an effective amount of oxygen is more apparent at 80 kV than at 60 kV, the latter of which the authors mainly used. Isn't the difference in the knock-on threshold between B and N even smaller at 60 kV? The reasons for the occurrence of circular pores in ultra high-vacuum conditions and how this phenomenon can be reconciled with the conventional knock-on threshold mechanism remain unclear.
At present, the discussion relies primarily on comparisons of cross-sections as a function of number of atoms, which may not be sufficient to explain this apparent difference. The authors could clarify whether the previously reported knock-on threshold values for hBN require revision for the related experiments. Further discussion would be valuable as the point is likely to raise questions.
Reference 1. Zobelli A, Gloter A, Ewels CP, Seifert G, Colliex C. Electron knock-on cross section of carbon and boron nitride nanotubes. Physical Review B 2007, 75(24): 245402.

2. According to the knock-on threshold for B and N, triangular pore formation appears to be more prevalent with continuous, uniform beam irradiation over a larger field of view in TEM mode with a parallel beam than with a scanning converged beam in STEM mode. I believe that a converged electron beam has a more pronounced drilling effect on the hBN specimens than a parallel beam does, and that it pumps more oxygen onto them. The authors should address this point.

3. The authors estimate the rate of molecular impingement using classical kinetic gas theory, which is commonly applied in vacuum systems. However, given that the experiments are performed within a TEM column, where electron beam effects may differ from ideal vacuum assumptions, it is unclear whether this work can be directly applied without modification. In this regard, Equation (4) may require further modification. The authors are encouraged to comment on the validity and limitations of this approach in the specific context of TEM experiments.

4. The results presented in Figure 5 are particularly interesting, as they suggest that pore growth depends not only on oxygen partial pressure but also on the electron flux density in magnified image, implying an interplay between oxygen availability and electron beam. However, although TEM instrumentation allows quantitative estimation of electron dose and dose rate, the discussion remains largely qualitative. The authors are encouraged to provide a more quantitative analysis of this correlation to strengthen the manuscript.

5. While the Nion STEM used in this study is known for the atomic spatial resolution, direct imaging or spectroscopic

evidence supporting the involvement of oxygen remains limited. The images provided in Supplementary Figure 1 do not appear sufficient to conclusively support the proposed role of oxygen. Given the capability for frame-by-frame analysis, the authors are encouraged to provide experimental evidence similar to prior studies that directly visualized oxygen-related processes in graphene systems.[Ref.2]
Reference 2. Ryu GH, Lee S, Kim JH, Lee GD, Lee Z. OH molecule-involved formation of point defects in monolayer graphene. Nanotechnology 2021, 32(2).

Minor comments
1. The experiments are conducted using a customized Nion TEM (Nion UltraSTEM 100 in Vienna) equipped with gas control capabilities. Since this constitutes a key instrument of the manuscript, the authors are encouraged to clarify how this setup differs from conventional TEM platforms (e.g., Thermo Fisher Scientific or JEOL etc.), and why such gas control is feasible in the present system. Although relevant references are cited, a concise and self-contained explanation within the manuscript would significantly improve clarity for readers unfamiliar with this instrumentation.

2. In the introduction, the importance of defect control in hBN could be written more clearly. In particular, from the perspective of the authors' suggestion of providing "a route toward atomically precise engineering", further explanation why and how control over pore morphology and edge termination is technologically or scientifically relevant would strengthen the manuscript.

3. On page 4 in the manuscript, the term "slightly hexagonal" is used. Given the significant geometric difference between circular and hexagonal shapes, the intended meaning should be clarified to avoid ambiguity.

4. Authors need to verify whether the interpretation of intensity values in the MAADF images presented in the Supplementary Information is consistent with the stated contrast mechanism.

Reviewer #2

(Remarks to the Author)
The authors present the results of an engaging study about pore formation in 2D hBN in the electron microscope, revealing that under UHV conditions circular pores are formed whereas upon introducing a small amount of O_2 into the sample chamber, preferential boron atom ejection emerges forming triangular pores with nitrogen edge termination. This is a significant finding, since for many years it has by most been assumed that nitrogen-terminated pores (in non UHV microscopes) were forming due to preferential knock-on displacement of the slightly lighter atom (boron). In contrast, the systemic experiments and supporting simulations of the present study show that chemical assist by residual oxygen in the column can explain the triangles, albeit the full mechanism behind the preferential boron atom removal is not yet fully understood. While investigations of pore growth in hBN and other 2D materials in the electron microscope have been ongoing for some while, the present work reveals new crucial insight that can be leveraged for deterministic atomic engineering to create pores of the desired shape and edge functionality for applications ranging from molecular sensing to single-atom catalysis.

I recommend the manuscript for publication, with just the following comments/suggestions:

In the Abstract, the authors state that "[…] even small amounts of oxygen in the atmosphere during the experiment change the pore shapes into triangles […]" This makes it sound a bit like a circular pore reconfigures to form a triangle shaped one – i.e. the atoms just rearrange without removal. To make the point clearer, perhaps wording like "drives the pores to grow into triangle shapes" may help.

In the Introduction, first sentence, hBN is described as being a wide bandgap insulator. I understand that later comparison between hBN's insulating behavior and the conductive behavior of graphene is made, but strictly speaking all insulators have wide bandgaps. I would opt for "wide bandgap semiconductor". Maybe further down, for consistency, one could say something like "relative insulators such as hBN".

In Figure 1 it looks like the fields of view are all the same. If so, this can be stated in the caption and the 2nm scale could, for example, be added to 1(f) (and removed from 1(d)) or just added to all images.

On Page 4, last paragraph, the authors say that "defects emerge and grow into pores". Since defects can also be substituted atoms, it would be helpful to say "vacancy defects", since presumably that is what is being referred to.

Figure 2: Again, are these the same FOV? One could just move the 0.05nm2 label in (b) to the left of the pore and add the scale marker there as well.

Figure 4: Same scale bar/FOV issue as above. Also, maybe colored rectangles or thicker lines would help highlight the marked regions.

Figure 3(a) would be easier to follow if the legend contained the full information about all the symbols used (rather than splitting this information between the legend and the caption). There is space to the left of the current legend, and if the plot started at x=0, then there would be more space underneath the present key. Either way, it would be helpful if the symbols could all be explicitly labeled (e.g. a column for 60kV and the filled shapes, and a column for 80kV and the open shapes). In

addition, in the caption, it would be helpful to write "(UHV)" next to 4x10-10mbar (since in (a) this is just labeled as UHV without the pressure value).

Page 9 discussion of Fig. 5: Perhaps safer to say "[…] atomic oxygen is presumably available […]".

In the Methods, one might infer that monolayers were exfoliated from the bulk crystal, since the wording "to exfoliate individual layers" was used. However, in Fig. 1, one sees that the exfoliated samples consisted (in this example) of 3 layers. Or at least, regions where e-beam exfoliation down to 3 layers and beyond are shown. The Methods section should thus be reworded to make it clear that the authors did not necessarily mechanically exfoliate to monolayer.

I congratulate the authors on their very thorough investigation!

Reviewer #3

(Remarks to the Author)
I co-reviewed this manuscript with one of the reviewers who provided the listed reports. This is part of the Nature Communications initiative to facilitate training in peer review and to provide appropriate recognition for Early Career Researchers who co-review manuscripts.

Reviewer #4

(Remarks to the Author)
The manuscript by Javed et al. on the "Origin of circular and triangular pores in electron-irradiated hexagonal boron nitride" reports observations on pore etching of hBN in the scanning transmission electron microscope.

The experimental conditions are reasonably well defined and the key observation, a significant increase of the etch rate and an effect on the pore shape, are convincingly demonstrated. The manuscript is in general well written and the data is carefully analyzed. There are some minor issues with the manuscript that can be removed by a revision.

Whether the manuscript is of sufficient relevance for publication in Nature communications is largely open to the referee. Although the authors tried their best, the manuscript lacks a convincing explanation for the preferential etching of B-edges by oxygen under irradiation conditions. On the positive side, the manuscript removes confusion in the field. Taken together, publication in Nature Communications can be justified, but the manuscript is certainly not a "must publish" manuscript.

The authors may want to consider the following issues prior to publication
- On page 2 the authors write: "However, the situation for hBN is not clear, since boron nitride nanotubes have been reported to be resistant to oxygen in the presence of an electron beam [17]" In reference 17 the reviewer reads: "This shows the damage rate has a dependency on the oxygen pressure." And from Figure 3 in reference 17 it is obvious that the electron dose to damage drops by an order of magnitude with increasing oxygen pressure during imaging. The authors should adjust their inadequate representation of previous work.

- It is common understanding that ultrahigh vacuum (UHV) starts below 1 x 10^-9 mbar pressure. The authors explicate that their pressure reading is an order of magnitude too small. Thus, the excessive use of the term UHV for the "pressure" of 4 x 10^-10 mbar which is in fact a high vacuum pressure of 4 x 10^-9 mbar is somewhat confusing. The reviewer suggests to just drop the claim of UHV.

- On page 8 the authors state: "…however, the smaller pores have remained practically unchanged, and only minor changes have occurred in the larger pores, presumably due to mechanical stress related to sample handling. This finding is in clear disagreement with Ref. [29], where the authors reported that pores in hBN grew and changed shape after one week of air exposure." Checking reference 29 the reviewer considers the results of the authors quite consistent with ref. 29. Upon air exposure also in the authors' experiments the flakes increased in size, may be somewhat less than in ref 29, but not in clear disagreement with ref. 29.

- Lastly, as the authors realized more or less themselves, the additional DFT calculations do not provide insight into the mechanism of etching. The much lower N-O desorption barrier in fact tends in the reviewer's opinion to suggest that N edges should etch preferentially. As a reader it is not clear that the authors do have an idea how to tackle the problem or which DFT results would have solved the problem of understanding the etching process and its dependence on oxygen. The reviewer suggests that the authors reconsider the value of their DFT calculations and the message they want to convey.

Version 1:

Reviewer comments:

Reviewer #1

(Remarks to the Author)
In response to the reviewers' comments, the manuscript has been significantly improved. The authors are commended for conducting additional experiments with a defocused probe and for providing a quantitative framework that directly supports the central hypothesis. They transparently acknowledge the remaining mechanistic uncertainties rather than overstating their conclusions. This lends additional credibility to their work and reflects a high standard of scientific rigor. It is hoped that the ongoing instrument upgrade will enable future investigations to elucidate the atomistic mechanism of oxygen-assisted boron removal further, answering an open and compelling question. The manuscript is well-written, the experimental design is thorough, and the central finding is convincingly demonstrated. This revision is suitable for publication in Nature Communications.

Reviewer #3

(Remarks to the Author)
I co-reviewed this manuscript with one of the reviewers who provided the listed reports. This is part of the Nature Communications initiative to facilitate training in peer review and to provide appropriate recognition for Early Career Researchers who co-review manuscripts.

Reviewer #4

(Remarks to the Author)
The authors responded adequately to the other reviewers and my own comments.
Therefore, now I recommend publication.

Open Access This Peer Review File is licensed under a Creative Commons Attribution 4.0 International License, which permits use, sharing, adaptation, distribution and reproduction in any medium or format, as long as you give appropriate credit to the original author(s) and the source, provide a link to the Creative Commons license, and indicate if changes were made.
In cases where reviewers are anonymous, credit should be given to 'Anonymous Referee' and the source.
The images or other third party material in this Peer Review File are included in the article’s Creative Commons license, unless indicated otherwise in a credit line to the material. If material is not included in the article’s Creative Commons license and your intended use is not permitted by statutory regulation or exceeds the permitted use, you will need to obtain permission directly from the copyright holder.
To view a copy of this license, visit https://creativecommons.org/licenses/by/4.0/

We appreciate receiving the reviewer comments on our manuscript, which we have taken account in this revision. Importantly, we have carried out additional experiments to provide a quantitative analysis of the influence of the oxygen atmosphere on the shape of pores forming under electron irradiation. Gladly, we were able to do this just before the microscope was decommissioned due to a major instrument upgrade, that is still taking place.

In the following, we provide a list of changes made to the manuscript and a point-by-point reply to all reviewer reports. In our view, the manuscript has further improved based on the reviewer comments, and we expect that it can be accepted for publication in its current form.

With best regards, on the behalf of all authors,
Univ.-Prof. Dr. Jani Kotakoski

List of changes to the manuscript

- Additional experimental data was collected with a defocused electron probe. Barbara Maria Mayer participated in these experiments, and has been added as an additional author.
- Several stylistic and typographic changes.
- Changes corresponding to the reviewer reports, as detailed below.

Reviewer #1 (Remarks to the Author)

The manuscript presents experimental evidence that oxygen radicals in the microscope column are responsible for the formation of triangular pores in hBN under electron irradiation. The systematic control of oxygen partial pressure and the quantitative analysis of edge atom removal probabilities represent clear strengths of the work. The results challenge a widely accepted interpretation in the previous articles. Nevertheless, several aspects of the mechanistic interpretation require clarification or further support before the conclusions can be considered robust. I list below several major points that should be addressed in a revised version.

Major comments

1. It is well known that under high-vacuum conditions in TEM, triangular hole formation with N-termination can be clearly observed because 80 kV lies between the knock-on threshold values of B (74 kV) and N (84 kV)1.[Ref.1] While the present manuscript tried to demonstrate that oxygen plays a key role in determining the pore shape, I think the difference between the presence and absence of an effective amount of oxygen is more apparent at 80 kV than at 60 kV, the latter of which the authors mainly used. Isn't the difference in the knock-on threshold between B and N even smaller at 60 kV? The reasons for the occurrence of circular pores in ultra high-vacuum conditions and how this phenomenon can be reconciled with the conventional knock-on threshold mechanism remain unclear.
At present, the discussion relies primarily on comparisons of cross-sections as a function of number of atoms, which may not be sufficient to explain this apparent difference. The authors could clarify whether the previously reported knock-on threshold values for hBN require revision for the related experiments. Further discussion would be valuable as the point is likely to raise questions.
Reference 1. Zobelli A, Gloter A, Ewels CP, Seifert G, Colliex C. Electron knock-on cross section of carbon and boron nitride nanotubes. Physical Review B 2007, 75(24): 245402.

Authors' reply:

We thank the reviewer for these questions, which we address in detail below.

Over the past years, it has been demonstrated (see Refs. 13, 23 and 24 of the manuscript) that the damage mechanism is more complicated than simple knock-on in 2D materials other than graphene, because of the contribution of electronic excitations. Therefore, it is not possible to predict the atom displacement using only the previously reported knock-on threshold values even under ultra-high vacuum (without the effect of atmosphere). In particular, Ref. [13; https://doi.org/10.1002/smll.202301926] provided the most precise cross-section measurements at UHV to date, showing that the probability of ejecting B and N is surprisingly similar both at 60 and 80 keV (Figure 6 of that article; the values with overlapping uncertainties are cited on page 6 of our manuscript).

By contrast, we observe at both voltages circular pores in ultra-high vacuum, and triangular ones under oxygen atmosphere. Regarding low-pressure experiments (below 1e-9 mbar), the effect of oxygen for the pore growth is indeed smaller at 80 kV as compared to 60 kV. However, at the highest pressure studied here, the damage rate is the same at both voltages, highlighting that under these conditions the atmosphere defines the damage rather than direct electron-beam effects.

We have improved this discussion in the manuscript (pages 3-4), which now reads:

"Formation of pores in hBN under electron irradiation is of particular interest due to their characteristic triangular shape, known for nearly two decades [17–21]. The broadly accepted explanation for this was that boron is easier to displace than nitrogen, owing to their different displacement cross sections resulting from their mass difference and differing displacement threshold energies [22]. However, these values correspond to knock-on damage only assuming an elastic scattering event, which has been shown to not fully describe electron beam damage in 2D materials that are not as good conductors as graphene [13, 23, 24]. Surprisingly, the most precise recently measured cross-sections for B and N ejection under both 60- and 80-keV irradiation are equal within the uncertainty of the experiment [13]. Both nitrogen and boron single vacancies [13], and even boron-terminated tetravacancies [6, 25], have been reported to form under electron irradiation at room temperature.

In these experiments, all larger structures have corresponded to triangular shapes with nitrogen-terminated edges. Non-triangular pores have only been reported when the electron beam was condensed to increase the current density to get hexagonal pores [26], or as a result of "drilling" with a focused 200-keV electron beam onto a few-layer hBN sample to get round pores [27]. However, at elevated temperatures, the picture is more complicated. For example, Cretu et al. [28] demonstrated that at 1200 ◦C hexagonal pores form, whereas at 800 ◦C they are circular. However, in that study, the pores were triangular at room temperature. While it has been speculated that the residual vacuum in the microscope could also play a role in the preferred removal of boron atoms [22, 26, 28], this hypothesis has not been tested due to the required customized experimental equipment."

2. According to the knock-on threshold for B and N, triangular pore formation appears to be more prevalent with continuous, uniform beam irradiation over a larger field of view in TEM mode with a parallel beam than with a scanning converged beam in STEM mode. I believe that a converged electron beam has a more pronounced drilling effect on the hBN specimens than a parallel beam does, and that it pumps more oxygen onto them. The authors should address this point.

We agree that the convergence of the electron beam plays a role here, as does the difference between a scanning probe and a stationary one. Therefore, we carried out new experiments with a defocused probe to confirm that our main findings also hold for a more parallel illumination and for a stationary electron beam.

The new results are shown in new Supplementary Figure 1. Here, the sample was irradiated with a defocus of 125 nm (illuminated area ca. 7.5 nm in diameter) at the pressure of 4e-9 mbar for around four minutes. Under these conditions, the pores were not triangular. Next, we increased the pressure to 1.3e-8 mbar, which provides ca. three times more many oxygen molecules impinging on the surface per time. Now, after four minutes of irradiation, the created pores were triangular.

Additional data was recorded with different defocus values, as shown in the new Supplementary Figure 2. We also included in the plot shown there data points related to experiments with the scanning focused probe. For all experiments, we calculated the number of oxygen molecules

impinging per unit area and per unit time and divided it by the corresponding number of electrons based on the beam current. As can be seen in the figure, there is a transition from circular to triangular pores at ca. 3 oxygen molecules per 10 million electrons (using the pressure from the objective area gauge to estimate the impingement rate). For a higher number of molecules, the pores are always triangular, and for a lower number they are not, with a tendency towards truly circular pores a the lowest number of molecules. We also added to the plot two examples from literature, where we had to estimate the oxygen partial pressure. In both cases, the results support our own findings.

Overall, the new experiments clearly support our hypothesis that the oxygen partial pressure is the key for the formation of nitrogen-terminated triangular pores in hBN under electron irradiation. Only when there are no sufficient number of oxygen molecules available, are the pores circular demonstrating that the cross sections for removing B and N atoms from pore edges are not sufficiently different to lead to a preferred edge termination.

These results are now discussed in the revised manuscript on page 10, which reads:

"This observation also holds for irradiation with a defocused electron probe. Indeed, it has been shown that when converging the beam into a smaller area, pores become nontriangular where they were triangular before focusing [26]. To avoid potential effects from the scanning and focused electron probe and to adjust the local current density, we also conducted experiments with a defocused probe. In oxygen pressure of 4×10^{-9} mbar and with a dose rate of ca. 1.7×10^{7} $e^{-}s^{-1}nm^{-2}$, we observe both triangular and round pores, showing that under these conditions the two mechanisms are equally prevalent. However, when the pressure is increased to 1.3×10^{-8} mbar while keeping the dose rate constant, the created pores are triangular. At a lower dose rate of ca. 1×10^{6} $e^{-}s^{-1}nm^{-2}$, pores remain triangular at both partial presures of 4×10^{-9} and 1.3×10^{-8} mbar. These experiments are summarized in Supplementary Figs. 1–2, and Supplementary Table 1 [37]. Together, these data allow us to estimate a quantitative correlation between the number of oxygen molecules and the number of electrons per area per time and the shape of the pores: when there are more than five oxygen molecules per ten million electrons, the pores that appear are triangular, whereas for fewer than three molecules, they are circular. In between these values, we find a mixture of shapes, including triangles, circles, and deformed hexagons."

3. The authors estimate the rate of molecular impingement using classical kinetic gas theory, which is commonly applied in vacuum systems. However, given that the experiments are performed within a TEM column, where electron beam effects may differ from ideal vacuum assumptions, it is unclear whether this work can be directly applied without modification. In this regard, Equation (4) may require further modification. The authors are encouraged to comment on the validity and limitations of this approach in the specific context of TEM experiments.

The assumption of kinetic gas theory is used here only to estimate the number of oxygen molecules impinging on the surface, which should to the first approximation be true in an electron microscope column. This is also reflected in our mass spectroscopy experiments that show that with and without the electron beam, the gas composition is the same – there are simply not enough electrons to appreciably alter the overall vacuum composition.

Where the electron beam does make a difference, however, is in breaking the oxygen molecules that land within the imaged area. After the molecules have been broken, the thus-created oxygen radical are expected to remain on the surface of the sample, migrate to defects (such as pore edges), and

cause structural changes there. In the context of this study, these changes are related to promoting the removal of B edge atoms, although the exact atomic-scale mechanism remains elusive.

We have clarified this now in the revised manuscript (page 10):

"To estimate the number of oxygen molecules at the surface at a given pressure, we used the kinetic theory of gases [38], which is typically used for vacuum systems. Although the microscope column has additionally an electron beam that interacts locally with the atmosphere and the sample, it has a negligible influence on the composition of gases. The rate of impingement in vacuum [39] can be calculated by…"

4. The results presented in Figure 5 are particularly interesting, as they suggest that pore growth depends not only on oxygen partial pressure but also on the electron flux density in magnified image, implying an interplay between oxygen availability and electron beam. However, although TEM instrumentation allows quantitative estimation of electron dose and dose rate, the discussion remains largely qualitative. The authors are encouraged to provide a more quantitative analysis of this correlation to strengthen the manuscript.

We thank the referee for pointing out the qualitative nature of the discussion in our original submission. As explained above, we have now improved on this using experiments with a defocused stationary probe that allowed us to provide a quantitative limit for the appearance of triangular pores.

5. While the Nion STEM used in this study is known for the atomic spatial resolution, direct imaging or spectroscopic evidence supporting the involvement of oxygen remains limited. The images provided in Supplementary Figure 1 do not appear sufficient to conclusively support the proposed role of oxygen. Given the capability for frame-by-frame analysis, the authors are encouraged to provide experimental evidence similar to prior studies that directly visualized oxygen-related processes in graphene systems.[Ref.2]
Reference 2. Ryu GH, Lee S, Kim JH, Lee GD, Lee Z. OH molecule-involved formation of point defects in monolayer graphene. Nanotechnology 2021, 32(2).

Unfortunately, as atomic-scale dynamics take in most cases place at picosecond time scales, this makes it impossible to image true dynamics at atomic resolution, where the typical frame time is a large fraction of a second. However, we do occasionally observe what looks like oxygen atoms embedded in small vacancy structures. An example of such an image was already shown (figure 7 in the revised manuscript), and has now been improved to better show the corresponding atomic intensities.

Minor comments

1. The experiments are conducted using a customized Nion TEM (Nion UltraSTEM 100 in Vienna) equipped with gas control capabilities. Since this constitutes a key instrument of the manuscript, the authors are encouraged to clarify how this setup differs from conventional TEM platforms (e.g., Thermo Fisher Scientific or JEOL etc.), and why such gas control is feasible in the present system. Although relevant references are cited, a concise and self-contained explanation within the manuscript would significantly improve clarity for readers unfamiliar with this instrumentation.

We have added additional information about the setup to the Methods section (Page 16) of the manuscript. This part reads now:

"The Vienna microscope has been modified to add extra pumping in the microscope column [32] and a leak valve that allows introducing gases into the objective area during imaging. Additionally, it is part of an interconnected system of vacuum instruments, allowing samples to be kept in ultra-high vacuum between experiments [48]. This also means that the microscope column is not exposed to the atmosphere during sample exchange, maintaining a base pressure in the microscope at the sample is around 10^{-10} mbar (as measured by the objective area gauge), which is around three orders of magnitude lower than typical microscopes. An equilibrium between leaking and pumping can be achieved to create a desired atmosphere up to ca. 4×10^{-6} mbar during atomic-resolution imaging [14]."

2. In the introduction, the importance of defect control in hBN could be written more clearly. In particular, from the perspective of the authors' suggestion of providing "a route toward atomically precise engineering", further explanation why and how control over pore morphology and edge termination is technologically or scientifically relevant would strengthen the manuscript.

We have added additional information about this to the introduction of the manuscript. The added text is:

"Pore shape directly influences the edge chemistry, and local electronic and transport properties [29]. Triangular pores provide anisotropic, while circular pores provide more isotropic interaction landscapes, which can affect DNA translocation and signal stability during sequencing [30], as well as selectivity and permeability in filtration [31]."

3. On page 4 in the manuscript, the term "slightly hexagonal" is used. Given the significant geometric difference between circular and hexagonal shapes, the intended meaning should be clarified to avoid ambiguity.

We have now reworded the description, which now reads:

"… and the pores appear round ~~or slightly hexagonal~~, in contrast to other reported room-temperature experiments…"

4. Authors need to verify whether the interpretation of intensity values in the MAADF images presented in the Supplementary Information is consistent with the stated contrast mechanism.

As noted above, we have improved on the presentation of the figure to show that the intensities indeed correspond to our interpretation.

Reviewer #2 (Remarks to the Author)

The authors present the results of an engaging study about pore formation in 2D hBN in the electron microscope, revealing that under UHV conditions circular pores are formed whereas upon introducing a small amount of O2 into the sample chamber, preferential boron atom ejection emerges forming triangular pores with nitrogen edge termination. This is a significant finding, since for many years it has by most been assumed that nitrogen-terminated pores (in non UHV microscopes) were forming due to preferential knock-on displacement of the slightly lighter atom (boron). In contrast, the systemic experiments and supporting simulations of the present study show that chemical assist by residual oxygen in the column can explain the triangles, albeit the full mechanism behind the preferential boron atom removal is not yet fully understood. While investigations of pore growth in hBN and other 2D materials in the electron microscope have been

ongoing for some while, the present work reveals new crucial insight that can be leveraged for deterministic atomic engineering to create pores of the desired shape and edge functionality for applications ranging from molecular sensing to single-atom catalysis.

I recommend the manuscript for publication, with just the following comments/suggestions:

We thank the reviewer for their contribution to improve our manuscript. We address point-by-point all the raised issues below.

In the Abstract, the authors state that "[...] even small amounts of oxygen in the atmosphere during the experiment change the pore shapes into triangles [...]" This makes it sound a bit like a circular pore reconfigures to form a triangle shaped one – i.e. the atoms just rearrange without removal. To make the point clearer, perhaps wording like "drives the pores to grow into triangle shapes" may help.

We have changed this passage as suggested.

In the Introduction, first sentence, hBN is described as being a wide bandgap insulator. I understand that later comparison between hBN's insulating behavior and the conductive behavior of graphene is made, but strictly speaking all insulators have wide bandgaps. I would opt for "wide bandgap semiconductor". Maybe further down, for consistency, one could say something like "relative insulators such as hBN".

This is a fair point. We made these suggested changes to the text.

In Figure 1 it looks like the fields of view are all the same. If so, this can be stated in the caption and the 2nm scale could, for example, be added to 1(f) (and removed from 1(d)) or just added to all images.

Thank you for pointing this out. We have added the explanation to the captions of the relevant figures and moved all scale bars in all figures to the last image.

On Page 4, last paragraph, the authors say that "defects emerge and grow into pores". Since defects can also be substituted atoms, it would be helpful to say "vacancy defects", since presumably that is what is being referred to.

Yes, this is correct. We have modified the text accordingly.

Figure 2: Again, are these the same FOV? One could just move the 0.05nm2 label in (b) to the left of the pore and add the scale marker there as well.

Yes. The figure has been modified accordingly.

Figure 4: Same scale bar/FOV issue as above. Also, maybe colored rectangles or thicker lines would help highlight the marked regions.

Also these issues have been addressed, and the rectangles have been made thicker.

Figure 3(a) would be easier to follow if the legend contained the full information about all the symbols used (rather than splitting this information between the legend and the caption). There is space to the left of the current legend, and if the plot started at x=0, then there would be more space underneath the present key. Either way, it would be helpful if the symbols could all be explicitly labeled (e.g. a column for 60kV and the filled shapes, and a column for 80kV and the open shapes). In addition, in the caption, it would be helpful to write "(UHV)" next to 4x10-10mbar (since in (a) this is just labeled as UHV without the pressure value).

All the suggested changes have been implimented.

Page 9 discussion of Fig. 5: Perhaps safer to say "[...] atomic oxygen is presumably available [...]".

We have also made this change.

In the Methods, one might infer that monolayers were exfoliated from the bulk crystal, since the wording "to exfoliate individual layers" was used. However, in Fig. 1, one sees that the exfoliated samples consisted (in this example) of 3 layers. Or at least, regions where e-beam exfoliation down to 3 layers and beyond are shown. The Methods section should thus be reworded to make it clear that the authors did not necessarily mechanically exfoliate to monolayer.

This is correct. We have added this detail to the Methods section. It now reads (in the Methods section at pages 15-16):

"The exfoliated sample had a minimum of three layers, as shown in Fig. 1. To reach the single layer, the electron beam was continuously scanned over the area until a monolayer region formed."

I congratulate the authors on their very thorough investigation!

We thank the reviewer 2 for their kind words.

Reviewer #3 (Remarks to the Author)

I co-reviewed this manuscript with one of the reviewers who provided the listed reports. This is part of the Nature Communications initiative to facilitate training in peer review and to provide appropriate recognition for Early Career Researchers who co-review manuscripts.

We thank the reviewer for their contribution.

Reviewer #4 (Remarks to the Author)

The manuscript by Javed et al. on the "Origin of circular and triangular pores in electron-irradiated hexagonal boron nitride" reports observations on pore etching of hBN in the scanning transmission electron microscope.

The experimental conditions are reasonably well defined and the key observation, a significant increase of the etch rate and an effect on the pore shape, are convincingly demonstrated. The manuscript is in general well written and the data is carefully analyzed. There are some minor issues with the manuscript that can be removed by a revision.

Whether the manuscript is of sufficient relevance for publication in Nature communications is largely open to the referee. Although the authors tried their best, the manuscript lacks a convincing explanation for the preferential etching of B-edges by oxygen under irradiation conditions. On the positive side, the manuscript removes confusion in the field. Taken together, publication in Nature Communications can be justified, but the manuscript is certainly not a "must publish" manuscript.

We thank the referee for their candid assessment of the merits of our manuscript. As also noted in the reply to the first reviewer, we have now improved on the quantitative analysis of the work. We

hope that in the current state, the manucript has moved at least somewhat towards "must publish" also for this reviewer.

The authors may want to consider the following issues prior to publication

- On page 2 the authors write: "However, the situation for hBN is not clear, since boron nitride nanotubes have been reported to be resistant to oxygen in the presence of an electron beam [17]" In reference 17 the reviewer reads: "This shows the damage rate has a dependency on the oxygen pressure." And from Figure 3 in reference 17 it is obvious that the electron dose to damage drops by an order of magnitude with increasing oxygen pressure during imaging. The authors should adjust their inadequate representation of previous work.

We apologize for not being sufficiently clear in this passage. We meant to note that the cited study shows that BN nanotubes are more resistant to oxygen in the microscopy column than C nanotubes. To avoid confusion, we have now deleted this sentence.

- It is common understanding that ultrahigh vacuum (UHV) starts below 1 x 10^-9 mbar pressure. The authors explicate that their pressure reading is an order of magnitude too small. Thus, the excessive use of the term UHV for the "pressure" of 4 x 10^-10 mbar which is in fact a high vacuum pressure of 4 x 10^-9 mbar is somewhat confusing. The reviewer suggests to just drop the claim of UHV.

We understand the point of the reviewer. However, the usage of the term "UHV" refers practically in all cases to the reading of the vacuum gauge. With this logic, our lowest-pressure experiments are carried out in UHV.

We only note the factor of 10, when we try to estimate the actual number of oxygen molecules impinging on the surface, in order to be as precise there as we can. We have now clarified this point in the manuscript, and hope that the reviewer agrees that this is the least confusing way to handle the issue.

We have now added in several places the mention, that the pressures discussed are those measured by the vacuum gauge. We also removed the factor from equation 4, since it is not used in the new analysis explained above in the response to reviewer 1 to allow comparison over different instruments. The passage explaining the factor now reads (page 10):

"For the estimate above, we have simply used the gauge pressure at the objective area to allow comparison across different instruments. However, we previously estimated that, for the instrument used in these experiments, the pressure at the sample is about 10 times higher due to the specific geometry of the sample stage [15]. The impinging rate at different oxygen partial pressures used in this study, taking into account the additional factor of ten, is shown in Supplementary Table 2 [37]."

- On page 8 the authors state: "...however, the smaller pores have remained practically unchanged, and only minor changes have occurred in the larger pores, presumably due to mechanical stress related to sample handling. This finding is in clear disagreement with Ref. [29], where the authors reported that pores in hBN grew and changed shape after one week of air exposure." Checking reference 29 the reviewer considers the results of the authors quite consistent with ref. 29. Upon air exposure also in the authors' experiments the flakes increased in size, may be somewhat less than in ref 29, but not in clear disagreement with ref. 29.

We need to point out that in Ref. 29, the change is said to be a "dramatic geometry change". Also, in Fig. 2 of Ref. 29, small triangle pores are shown to have merged into big triangle pores with just 1 h

exposure to air. Furthermore, in Fig. 3 multilayer hBN is kept for 1 week in air, and as a result the pores are unrecognizable, and most of the edges are eroded. Therefore, we find that it is justified to point out that our results differ from those shown in Ref. 29.

However, perhaps the wording was too strong. We have now changed this sentence to (pages 8-9):

"… handling. However, it has previously been reported that air exposure can lead to a dramatic geometry change in hBN pores [33]. Our results suggest that such a geometry change may be due to an additional process and not the air exposure alone."

- Lastly, as the authors realized more or less themselves, the additional DFT calculations do not provide insight into the mechanism of etching. The much lower N-O desorption barrier in fact tends in the reviewer's opinion to suggest that N edges should etch preferentially. As a reader it is not clear that the authors do have an idea how to tackle the problem or which DFT results would have solved the problem of understanding the etching process and its dependence on oxygen. The reviewer suggests that the authors reconsider the value of their DFT calculations and the message they want to convey.

The reviewer correctly points out that the DFT calculation results are inconclusive with respect to the actual etching mechanism. They suggest that O preferentially binds to B atoms at pore edges, but do not show a clear pathway for the etching process itself, especially since the N-O desorption barrier is lower, as the reviewer points out.

While we tried to be as open about this as possible already in the original submission, we have now further pointed this out by modifying the corresponding text on pages 13-14. The last paragraph describing the simulations now reads:

"Overall, these simulation results do not provide a complete picture of pore formation in hBN under electron irradiation in a low-pressure atmosphere. There are several possible explanations for this. Firstly, the experimental situation may be more complex than what is captured by the simulations, for example, due to the diffusion dynamics of oxygen radicals on the basal plane of hBN as they approach the pore edge, or structural changes caused by oxygen adsorption near the edge. Secondly, electron-beam-induced damage in semiconducting and insulating 2D materials has not been satisfactorily explained yet, even for pristine materials, despite recent efforts [23, 24, 43, 44]."
